# Mueller-matrix imaging polarimetry elevated by wavelet decomposition and polarization-singular processing for analysis of specific cancerous tissue pathology

**DOI:** 10.1117/1.JBO.28.10.102903

**Published:** 2023-07-08

**Authors:** Anton Sdobnov, Volodymir A. Ushenko, Liliya Trifonyuk, Oksana Bakun, Marta Garazdyuk, Irina V. Soltys, Olexander Dubolazov, Olexander G. Ushenko, Yuriy A. Ushenko, Alexander Bykov, Igor Meglinski

**Affiliations:** a University of Oulu, Opto-Electronics and Measurement Techniques, Oulu, Finland; b Chernivtsi National University, Optics and Publishing Department, Chernivtsi, Ukraine; c Rivne State Medical Center, Rivne, Ukraine; d Zhejiang University, Taizhou Research Institute, College of Electrical Engineering, Hangzhou, China; e Aston University, College of Engineering and Physical Sciences, Birmingham, United Kingdom

**Keywords:** polarized light, Mueller-matrix, imaging polarimetry, birefringence, cancer, polarization-singular, wavelet decomposition

## Abstract

**Significance:**

Mueller-matrix polarimetry is a powerful method allowing for the visualization of malformations in biological tissues and quantitative evaluation of alterations associated with the progression of various diseases. This approach, in fact, is limited in observation of spatial localization and scale-selective changes in the poly-crystalline compound of tissue samples.

**Aim:**

We aimed to improve the Mueller-matrix polarimetry approach by implementing the wavelet decomposition accompanied with the polarization-singular processing for express differential diagnosis of local changes in the poly-crystalline structure of tissue samples with various pathology.

**Approach:**

Mueller-matrix maps obtained experimentally in transmitted mode are processed utilizing a combination of a topological singular polarization approach and scale-selective wavelet analysis for quantitative assessment of the adenoma and carcinoma histological sections of the prostate tissues.

**Results:**

A relationship between the characteristic values of the Mueller-matrix elements and singular states of linear and circular polarization is established within the framework of the phase anisotropy phenomenological model in terms of linear birefringence. A robust method for expedited (up to ∼15  min) polarimetric-based differential diagnosis of local variations in the poly-crystalline structure of tissue samples containing various pathology abnormalities is introduced.

**Conclusions:**

The benign and malignant states of the prostate tissue are identified and assessed quantitatively with a superior accuracy provided by the developed Mueller-matrix polarimetry approach.

## 1 Introduction

In terms of biomedical optical imaging, the biological tissues are conditionally divided into two major groups.^1^ The first group contains tissues that highly scatter the light, e.g., skin, brain, sclera, blood, and vessel wall, whereas the second one consists of weakly scattering or nearly transparent tissues, such as cornea, eye lens, and thin histological sections of various types of biological tissues. Therefore, the optical properties of tissues in these groups are based, respectively, on multiple scattering (diffusion) approximation and single scattering. Although the approximation of photon diffusion is a cornerstone of optical imaging and near-infrared spectroscopy,^2^ it struggles to describe properly the propagation of polarized light in biological tissues both in single and multiple scattering regimes. In addition, neither single scattering nor multiple scattering approximations are not able to take into account the vector nature of the incident polarization and/or scattered light waves.

The use of polarized light as an “instrumental probe” allows for assessing quantitatively optical anisotropy of the poly-crystalline structure of biological fluids and tissues.^3^^–^^9^ The polarization introscopy approach is well developed and known as Mueller-matrix (MM) microscopy in transmitted mode.^10^^–^^15^ In fact, MM microscopy is an example of a successful synthesis of instrumental imaging polarimetry, diverse theoretical modeling, and image processing, utilizing the regression model of optical anisotropy,^11^ logarithmic MM decomposition,^12^^–^^15^ Monte Carlo-based assessment of polarized light conversion,^14^ and statistical analysis of MM images and optical anisotropy maps.^11^^,^^15^ A considerable result of biological tissue screening, obtained with the MM microscopy, is highly promising for the clinical application and pre-clinical studies of the poly-crystalline structure of biological tissues.^15^ In particular, the possibility of obtaining quantitative optical metrics to characterize the evolution of gastric tissue from a healthy state through inflammation to cancer using MM microscopy of gastric biopsies, a regression model of optical anisotropy, and statistical analysis of the obtained images has been demonstrated.^11^ Extension of the applied functional capabilities of MM microscopy ensured the application of the differential MM formalism in the analysis of experimental data.^12^^,^^13^^,^^15^ On this basis, the maps of depolarization and polarization of fixed uncolored histological sections of human skin tissues were obtained. This allowed for mitigating the influence of tissue slice thickness variations and increasing the contrast of polarimetric images for tissue diagnosis. In addition, the use of MM microscopy data in combination with logarithmic decomposition and polarization Monte Carlo simulation (within the framework of Mie theory approximation) opens a way for qualitative and quantitative analysis of thin tissue sections to extract information about tissue microstructure,^15^^,^^16^ which is not available in conventional microscopy.

It should be noted that the analyzed polarization introscopy methods^3^^–^^6^^,^^8^^,^^9^^,^^17^^,^^18^ and MM^10^^–^^16^ microscopy facilitate obtaining and visual analysis of the topological and coordinate structure of optical anisotropy maps of biological preparations. However, such analysis is somewhat subjective and does not provide a quantitative (objective) assessment of the severity or stages of pathology. Therefore, it is relevant to obtain a set of additional perceptible objective criteria (e.g., such as statistical moments of the first to fourth orders^11^^,^^15^) for MM characterization and differentiation of pathological conditions that are coordinate (x,y) and scale (a) localized in the biological tissue layer. However, the topological information about the optical anisotropy of the biological layer appears to be integrally averaged over all coordinates and geometric dimensions of MM map images within a quantitative statistical analysis. For statistical quantification of polarization-detected local variations in optical anisotropy parameters, statistical analysis of scale-selective samples from MM data derived from polarization-singular^19^^–^^34^ and scale-selective^35^^–^^40^ wavelet analysis may be most appropriate.

The use of the polarization-singular approach defines the lines/surfaces at each point of a polarization-inhomogeneous field with indefinite (singular) parameters.^19^^–^^22^^,^^41^ These points are as follows:

(1) “C” states are the points of circular polarization of the field, where the polarization ellipse degenerates into a circle and, accordingly, the direction of the main axis (azimuth) of the polarization ellipse is uncertain;
(2) “L” states are the points with linear polarization degenerated in the direction of the rotation of the electric vector.

The basic principles of complex vector singular analysis are formulated and described in details elsewhere,^19^^,^^22^^–^^26^ considered for optical fields,^27^^–^^29^ and practically implemented in biomedical imaging.^30^^–^^34^ Thus, for the first time, to our knowledge, biological tissues with a presence of linearly birefringence were characterized analytically in terms of the formation of linear and circular polarization singularities.^27^^–^^29^ A significant predominance of L states in comparison with C states is observed due to a more complex formation of circular polarization states and the domination of optically isotropic constituents within biological tissue morphology. The characteristic values of the fourth parameter of the Stokes vector were used as markers of polarization-singular states: $S_{4}=0$ for L and $S_{4}=\pm 1$ for ±C, whereas the distribution of polarization-singular states numbers (N(L), N(C)) was utilized to analyze images of biological tissue and fluid samples. It has been demonstrated that the third ($Z_{3}$) and fourth ($Z_{4}$) statistical moments characterizing the asymmetry and excess of the distributions $N(S_{4}=0)$ and $N(S_{4}=\pm 1)$ of singular points are sensitive to pathological changes in the poly-crystalline component of histological sections of biological tissues as well as in the poly-crystalline films of biological fluids. Such changes can lead to the necrosis of biological tissue morphological structure. As a result, the level of optical anisotropy decreases as well as the probability of $S_{4}=\pm 1$ for ±C formation. Quantitatively, this leads to an increase in the value of statistical moments $Z_{3}$ and $Z_{4}$.

The wavelet analysis is one of the main analytical methods for scale-selective estimation of line (1,2…,(n−1),n) pixel distributions q(x) of azimuth α, ellipticity β, and elements $f_{ik}$ of MM {F}.^35^^,^^36^ Utilizing the wavelet function, the distribution is expanded by the following equation:

$$q(x)=\overset{\infty }{\underset{a,b=-\infty }{\sum }}Q_{ab}\Omega_{ab}(x),$$ (1)

where $\Omega_{ab}(x)=\Omega (ax-b)$ is the basic function formed from the prototype function by offset b and scaling a, and the coefficients of this expansion are determined as

$$Q_{ab}=\int q(x)\Omega_{ab}(x)dx.$$ (2)

The wavelet transform allows for revealing both low-frequency and high-frequency characteristics of the distribution on the different coordinate scales (so-called “mathematical microscope”). Continuing the analogy with a mathematical microscope, the shift parameter b fixes the focal point of the microscope, the scale factor a shows the magnification, and the choice of the base wavelet Ω is the optical properties of the microscope. In this study, the second derivative of the Gaussian function–MHAT wavelet is used. Such a function has a narrow energy spectrum and two moments equal to zero (zero and first) that suit well for the analysis of complex signals:^37^^–^^40^

$$\Omega (t)=\frac{d^{2}}{dt^{2}}e^{-t^{2}/2}=(1-t^{2})e^{-t^{2}/2}.$$ (3)

The wavelet transforms of the one-dimensional distribution q(x) result in a two-dimensional array Q(a,b) of amplitudes. The distribution of these values in space (a is the spatial scale, and b is the spatial coordinate or localization) gives the information about the evolution of the relative contribution of components of different scales to the distribution under consideration and is called the spectrum of wavelet coefficients Q(a,b):

$$Q(a,b)=\frac{1}{{\left|a\right|}^{1/2}}\int_{-\infty }^{+\infty }q(x)\Omega (\frac{t-b}{a})dt.$$ (4)

The approbation of this approach demonstrates a significant improvement in the sensitivity and accuracy of MM polarimetry in the differential diagnosis of inflammatory and oncological conditions.^30^^–^^36^^,^^42^^,^^43^ However, the polarization-singular^19^^–^^29^^,^^41^^,^^44^^,^^45^ and scale-selective wavelet^37^^–^^40^^,^^46^^,^^47^ approaches in biomedical diagnosis require further developments.

This study is aimed at identifying the analytical relationship between the polarization-singular states of the object field of optically thin (non-depolarizing) layers obtained from biological tissues and the characteristic values of their MM images registered in transmitted light. A robust method for expedited (up to 15 min) polarimetric-based differential diagnosis of local variations in the poly-crystalline structure of tissue samples containing various pathology abnormalities is presented.

## 2 Materials and Methods

### 2.1 MM Imaging and Singular States

Determination of analytical relationships between the characteristic values of MM elements $f_{ik}$ of the biological layer and polarization-singular states of its object field is based on using linear birefringence approximation for single light scattering by fibrillary networks of optically thin (attenuation coefficient τ≤0.01÷0.02) layers of biological tissue.

According to the birefringence model of spatially structured fibrillar networks,^48^^–^^54^ MM is presented by the following expression:^17^^,^^18^

$$\left\{ F\}=\|\begin{array}{cccc} 1 & 0 & 0 & 0\\ 0 & f_{22} & f_{23} & f_{24}\\ 0 & f_{32} & f_{33} & f_{34}\\ 0 & f_{42} & f_{43} & f_{44} \end{array}\|,\right.$$ (5)

where

$$f_{ik}=\{\begin{array}{l} f_{22}=\cos^{2}\;2\rho +\sin^{2}\;2\rho \;\cos\;\delta ,\\ f_{23}=f_{32}=\cos\;2\rho \;\sin\;2\rho (1-\cos\;\delta ),\\ f_{33}=\sin^{2}\;2\rho +\cos^{2}\;2\rho \;\cos\;\delta ,\\ f_{42}=-f_{24}=\sin\;2\rho \;\sin\;\delta ,\\ f_{34}=-f_{43}=\cos\;2\rho \;\sin\;\delta ,\\ f_{44}=\cos\;\delta . \end{array}$$ (6)

Here, ρ is the direction of the optical axis, determined by the orientation of the fibril position in the plane of the biological layer; $\delta =\frac{2\pi }{\lambda }\Delta nl$ defines the phase shift between linearly orthogonal polarized components of the laser beam amplitude; λ is the wavelength; Δn characterises birefringence; and l is the geometric layer thickness.

Based on Eqs. (5) and (6), it is possible to determine the diagnostically important relationship between the characteristic values of MM elements and the formation conditions of “L” and “A^±C” polarization-singular states formed by a birefringent fibrillar network (Table 1).

**Table 1:** Relationships between the characteristic values of the elements and azimuthal invariants of MM and polarization singularities.

$f_{ik}$		“L”-point (δ=0,δ=π)	“+C”-point (δ=+π/2)	“-C”-point (δ=−π/2)
$f_{22}$	0	—	+(ρ=+π/4)	+(ρ=−π/4)
	1	+(δ=0)	—	—
	−1	+(δ=π)	—	—
$f_{23}=f_{32}$	0	+(ρ=±π/4)	—	—
	1	—	+(ρ=+π/4)	+(ρ=−π/4)
	−1	—	+(ρ=−π/4)	+(ρ=+π/4)
$f_{24}=-f_{42}$	0	+(ρ=0,π)	—	—
	1	—	+(ρ=+π/4)	+(ρ=−π/4)
	−1	—	+(ρ=−π/4)	+(ρ=+π/4)
$f_{33}$	0	—	+(ρ=0)	+(ρ=0)
	1	+(δ=0)	—	—
	−1	+(δ=π)	—	—
$f_{34}=-f_{43}$	0	+(ρ=±π/4)	—	—
	1	—	+(ρ=0)	+(ρ=+π/2)
	−1	—	+(ρ=+π/2)	+(ρ=0)
$\mathrm{MMI}(f_{ik})$	—	“L”-point	“+C”-point	“-C”-point
	—	(δ=0,δ=π)	(δ=+π/2)	(δ=−π/2)
$f_{44}=\cos\;\delta$	0	—	+(ρ=0÷π)	+(ρ=0÷π)
	1	+(δ=0)	—	—
	−1	+(δ=π)	—	—
$F_{22;33}\equiv (f_{22}+f_{33})-1=\cos\;\delta$	0	—	+(ρ=0÷π)	+(ρ=0÷π)
	1	+(δ=0)	—	—
	−1	+(δ=π)	—	—
$F_{42;43}\equiv \sqrt{\left(f_{42}^{2}+f_{43}^{2}\right)}=\sin\;\delta$	0	+(δ=0,δ=π)	—	—
	1	—	+(ρ=0÷π)	+(ρ=0÷π)
	−1	—	+(ρ=0÷π)	+(ρ=0÷π)
$F_{24;34}\equiv \sqrt{\left(f_{24}^{2}+f_{34}^{2}\right)}=\sin\;\delta$	0	+(δ=0,δ=π)	—	—
	1	—	+(ρ=0÷π)	+(ρ=0÷π)
	−1	—	+(ρ=0÷π)	+(ρ=0÷π)

In fact, most of the matrix elements $f_{ik}$ presented in Table 1 are azimuthally dependent on the magnitude of the rotation of the sample plane relative to the irradiation direction.^16^^–^^18^ Therefore, for representative groups of biological tissue samples, it is necessary to use other azimuthally invariant MM functionals, which are also presented in Table 1.

Using the information presented in Table 1, it is possible to determine a complete set of “A^±C”-points $\left(\delta =\pm \frac{\pi }{2}\right)$ on the image of a biological object. The coordinate position of each point, in this way, corresponds to the conditions $f_{44}=f_{22}=f_{33}=0$. Also, it is possible to determine a complete set of “L”-points (δ=0) on the polarization image with arbitrary azimuths (0≤ρ≤π). Here, each point corresponds to the conditions $f_{22}=f_{33}=1$. Finally, the “orthogonal” L-points are determined. The formation of each L-point is associated with the orthogonal orientations of the optical axes of birefringent fibrils as

$$\left\{ \begin{array}{llll} f_{34}=0, & L_{45,-45}-\text{points} & \text{for} & \rho =\pm \frac{\pi }{4}\\ f_{34}=\pm 1, & L_{0,90}-\text{points} & \text{for} & \rho =0;\frac{\pi }{2} \end{array}.\right.$$ (7)

For the “azimuthal-invariant” polarization-singular states

$$\left\{ \begin{array}{llll} f_{44};F_{22;33}=\pm 1, & L-\text{points} & \text{for} & \delta =\pi k,k=0;1;\ldots ;0\leq \rho \leq \pi \\ f_{44};F_{22;33}=0, & C-\text{points} & \text{for} & \delta =0.5\pi (2\;k+),k=0;1;\ldots ;0\leq \rho \leq \pi \\ F_{42;43;24;34}=0, & L-\text{points} & \text{for} & \delta =\pi k,k=0;1;\ldots ;0\leq \rho \leq \pi \\ F_{42;43;24;34}=\pm 1, & C-\text{points} & \text{for} & \delta =0.5\pi (2\;k+),k=0;1;\ldots ;0\leq \rho \leq \pi \end{array}.\right.$$ (8)

### 2.2 Tissue Samples

To validate the proposed method and show the possibility of determining the different types of tumors, the histological sections of prostate and uterine tissue were obtained using microtome with rapid freezing after radical prostatectomy. Four representative groups of obtained histological biopsy sections of tumors were formed: group 1:– n=36 adenoma samples; group 2: n=36 moderately differentiated (3+3 on Gleason’s pattern scale) carcinoma samples; group 3: n=36 myoma samples; and group 4: n=36 uterine endometriosis samples.

For the histological analysis, each tissue sample was first fixated with formalin (40% formaldehyde aqueous solution). After 24 h, the sample was washed with running water. Further, the sample was placed in alcohol with increasing concentrations (from 70% to 100%) to achieve tissue dehydration. After dehydration, the sample was fixated in a xylol-paraffin mixture for 1 to 2 h at a temperature of 52°C to 56°C. After that, the histological sections were cut using the standard microtome. Each section was further stained with hematoxylin-eosin. Further, the obtained sections were investigated by microscope and the position of the tumor was determined according to Gleason’s scale.

Table 2 presents the optical and geometric parameters of the obtained histological sections of prostate tumor biopsies from both groups.

**Table 2:** Optical and geometric parameters of histological sections of prostate (groups 1 and 2) and uterine (groups 3 and 4) tissues.

Parameter	Group 1	Group 2	Group 3	Group 4
Geometrical thickness h (μm)	20±0.15	20±0.15	20±0.15	20±0.15
Attenuation (extinction) coefficient τ, ${(\mathrm{cm}}^{-1})$	0.026±0.0014	0.028±0.0016	0.021±0.0011	0.022±0.0013

The geometric thickness of histological sections of prostate and uterine tissues was determined by the standard values of scale of the freezing microtome. Variations in geometric thickness h, μm within the plane of the histological sections (7  mm×7  mm) did not exceed ±0.15  μm and did not result in a significant change in optical thickness and single-scattering conditions.

The measurement of the extinction coefficient of the prostate tissue samples was carried out according to the standard procedure of light attenuation measurement^55^ using an integral light scattering sphere.^56^ The sample preparation procedure was conducted in accordance with the principles of the Declaration of Helsinki and in compliance with the International Conference on Harmonization-Good Clinical Practice and local regulatory requirements. The study was reviewed and approved by the appropriate Independent Ethics Committees, and written informed consent was obtained from all subjects prior to the study initiation.

### 2.3 Experimental Setup

**Fig. 1:**
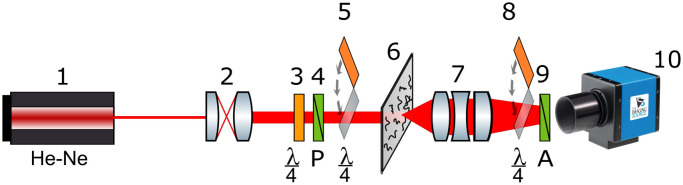
Experimental setup. (1) He–Ne laser, (2) collimator, (3) stationary quarter-wave plate, (5), (8) mechanically movable quarter-wave plates, (4), (9) polarizer and analyzer, (6) histological section, (7) polarizing microobjective, (10) CCD camera.

The experimental set up and the protocol of measurements of spatial distributions of the parameters of the Stokes vector and the elements of MM were developed earlier.^30^^,^^31^^,^^48^^,^^49^ Briefly, the optical setup is shown in Fig. 1. The setup utilized an He–Ne laser (Edmund Optics) emitting low-intensity (W=5.0  mW) light at 633 nm. The light beam was further collimated and passed through the quarter-wave plate (Achromatic True Zero-Order Waveplate, APAW 15 mm, Astropribor, Ukraine) and polarizer (B+W XS-Pro Polarizer MRC Nano, Kaesemann, Germany). After that, the light beam was passed through the sample and projected to the CCD-camera (1280×960  pixels, DMK 41AU02.AS, The Imaging Source, Germany) using a polarization microobjective (CFI Achromat P, focal length: 30 mm, numerical aperture: 0.1, increase: 4×, Nikon, Japan). Additional quarter-wave plates before the sample and after the microobjective were used for image analysis.

For the series of linear (0 deg, 90 deg, 45 deg) and right- ⊗ circular polarized illuminating laser beams, the Stokes-vector parameter $SV_{\left(i=1;2;3;4\right)}^{\left(0;45;90;⊗\right)}$ was defined for each pixel (m×n) as

$$\left\{ \begin{array}{c} SV_{\left(i=1\right)}^{\left(0;45;90;⊗\right)}(m\times n)=(I_{0}^{\left(0;45;90;⊗\right)}+I_{90}^{\left(0;45;90;⊗\right)})(m\times n);\\ SV_{\left(i=2\right)}^{\left(0;45;90;⊗\right)}(m\times n)=(I_{0}^{\left(0;45;90;⊗\right)}-I_{90}^{\left(0;45;90;⊗\right)})(m\times n);\\ SV_{\left(i=3\right)}^{\left(0;45;90;⊗\right)}(m\times n)=(I_{45}^{\left(0;45;90;⊗\right)}+I_{135}^{\left(0;45;90;⊗\right)})(m\times n);\\ SV_{\left(i=1\right)}^{\left(0;45;90;⊗\right)}(m\times n)=(I_{⊗}^{\left(0;45;90;⊗\right)}+I_{⊕}^{\left(0;45;90;⊗\right)})(m\times n). \end{array}\right.$$ (9)

Here, $I_{0;45;90;135;⊗;⊕}^{0;45;90;⊗}$ is the intensities of linearly (0 deg; 90 deg; 45 deg; 135 deg), right- (⊗) and left- (⊕) circularly polarized components of the filtered by means of polarizer 9 and quarter-wave plate 8 laser light. Finally, MM invariants were calculated as

$$f_{11}(m\times n)=0.5(SV_{1}^{0}+SV_{1}^{90})(m\times n);\;f_{12}(m\times n)=0.5(SV_{1}^{0}-SV_{1}^{90})(m\times n);\;f_{13}(m\times n)=(SV_{1}^{45}-f_{11})(m\times n);\;f_{14}(m\times n)=(SV_{1}^{⊗}-f_{11})(m\times n);\;f_{21}(m\times n)=0.5(SV_{2}^{0}+SV_{2}^{90})(m\times n);\;f_{22}(m\times n)=0.5(SV_{2}^{0}-SV_{2}^{90})(m\times n);\;f_{23}(m\times n)=(SV_{2}^{45}-f_{21})(m\times n);\;f_{24}(m\times n)=(SV_{2}^{⊗}-f_{21})(m\times n);\;f_{31}(m\times n)=0.5(SV_{3}^{0}+SV_{3}^{90})(m\times n);\;f_{32}(m\times n)=0.5(SV_{3}^{0}-SV_{3}^{90})(m\times n);\;f_{33}(m\times n)=(SV_{3}^{45}-f_{31})(m\times n);\;f_{34}(m\times n)=(SV_{3}^{⊗}-f_{31})(m\times n);\;f_{41}(m\times n)=0.5(SV_{4}^{0}+SV_{4}^{90})(m\times n);\;f_{42}(m\times n)=0.5(SV_{4}^{0}-SV_{4}^{90})(m\times n);\;f_{43}(m\times n)=(SV_{4}^{45}-f_{41})(m\times n);\;f_{44}(m\times n)=(SV_{4}^{⊗}-f_{41})(m\times n).$$ (10)

### 2.4 Methods of MMI Processing

#### 2.4.1 Linear scanning

To obtain objective criteria for MM polarization-singular differentiation between optical properties of prostate adenoma, carcinoma, and myoma-endometriosis samples, the following procedure was performed. MM images of the element $f_{44}(x,y)$ and a set of other MM invariants (see Table 1) were measured sequentially, and the coordinate grids of characteristic values were determined [Eq. (8)]. For example, matrix element $f_{44}(x,y)=\pm 1↔\prime \prime L\prime \prime -\text{point}$ and $f_{44}(x,y)=0↔\prime \prime \pm C\prime \prime -\text{point}$. By linear Oχ scanning along the m−th(m1,m2,….,mn) pixel row of the photosensitive pad $\left([\begin{array}{c} 111\;\ldots \;1n\\ \vdots \;\ddots \;\vdots \\ m1\;\ldots \;mn \end{array}]\right)$ of digital camera 10 for each individual pixel (mj), the number ($N_{m}j$) of characteristic values $f_{4}4$ within the corresponding column $\left(\begin{array}{c} 1j\\ .\\ .\\ mj \end{array}\right)$, and so on, is determined. Further, sets of linear dependencies of points for MM image characteristic values were determined in two orthogonal directions as $\left\{ \begin{array}{c} N((f_{44}=\pm 1),x)\equiv N(L,x);\\ N((f_{44}=\pm 1),y)\equiv N(L,y) \end{array}\right.$ and $\left\{ \begin{array}{c} N((f_{44}=0),x)\equiv N(C,x);\\ N((f_{44}=0),y)\equiv N(C,y). \end{array}\right.$ Similarly for other MMIs, there were $\left\{ \begin{array}{c} N((F_{22;33}=\pm 1),x)\equiv N(L,x);\\ N((F_{22;33}=\pm 1),y)\equiv N(L,y) \end{array}\right.$ and $\left\{ \begin{array}{c} N((F_{22;33}=0),x)\equiv N(C,x);\\ N((F_{22;33}=0),y)\equiv N(C,y). \end{array}\right.$, as well as $\left\{ \begin{array}{c} N((F_{42;43;24;34}=0),x)\equiv N(L,x);\\ N((F_{42;43;24;34}=0),y)\equiv N(L,y) \end{array}\right.$ and $\left\{ \begin{array}{c} N((F_{42;43;24;34}=\pm 1),x)\equiv N(C,x);\\ N((F_{42;43;24;34}=\pm 1),y)\equiv N(C,y). \end{array}\right.$

#### 2.4.2 Wavelet analysis

Then, wavelet analysis of linear dependencies N(L,x), N(C,x) was carried out, and two-dimensional arrays of wavelet coefficients $Q_{a,b}(L)=\int N(L,x)\Omega_{a,b}dx$ and $Q_{a,b}(C,x)=\int N(L,x)\Omega_{a,b}dx$ were determined. For the various scales $a_{j}=A$ of the MHAT function $\Omega_{(}a,b)$, sets of linear dependences of the wavelet coefficients amplitudes $Q_{A}(L,b)$ and $Q_{A}(C,b)$ were determined. For each scale $a_{j}=A$, the central statistical moments of the first and second orders $Z_{i=1;2}$^48^ were calculated, characterizing the average M and dispersion D of the distributions $Q_{A}(L,b)$ and $Q_{A}(C,b)$. Further, the step of large-scale “macro” scanning ($a_{j}^{\max}=10$) of MHAT function $\Omega_{(}a,b)$ was selected. The difference between the values of each central statistical moments of the first and second orders was calculated $\left(\Delta Z_{i=1;2}){)}_{k}=Z_{i=1;2}(a_{j+1}^{\max})-Z_{i=1;2}(a_{j}^{\max}\right)$. Then, the scale interval $\Delta a^{*}=(a_{j+1}^{\max}÷a_{j}^{\max})$ was determined, within the monotonic increase in the value $(\Delta Z_{i=1;2}{)}_{k}=Z_{i=1;2}(a_{j+1}^{\max})-Z_{i=1;2}(a_{j}^{\max})⩽0$ stops. Also, within limits $\Delta a^{*}$, a new series of values $\Delta Z_{i}=Z_{i}(a_{q+1}^{\min})-Z_{i=1;2}(a_{q}^{\min})$ was calculated with a step of discrete scaled “micro” scan $a_{q}^{\min}=2$. Then, the optimal scale $A^{*}$ was determined following the condition $\Delta Z_{i}(A^{*})=\max$. The mean $\overset{¯}{M}$, $\bar{D}$ and standard deviations $\sigma (\bar{M})$, $\sigma (\overset{¯}{D})$ were determined within the representative samplings of histological sections from group 1 to group 2 and group 3 to group 4.

#### 2.4.3 Informational analysis

To differentiate pathological states of prostate and uterine, for each statistical moment $Z_{i}$, the sensitivity (Se=(p/(p+g))100%), specificity (Sp=(c/(c+d))100%), and balanced accuracy (Ac=0.5(Se+Sp)) were calculated.^57^ Here, p and g are the numbers of correct and incorrect diagnoses, respectively, within group 2 and group 4; and c and d are the same within control group 1 and group 3.

## 3 Results and Discussion

**Fig. 2:**
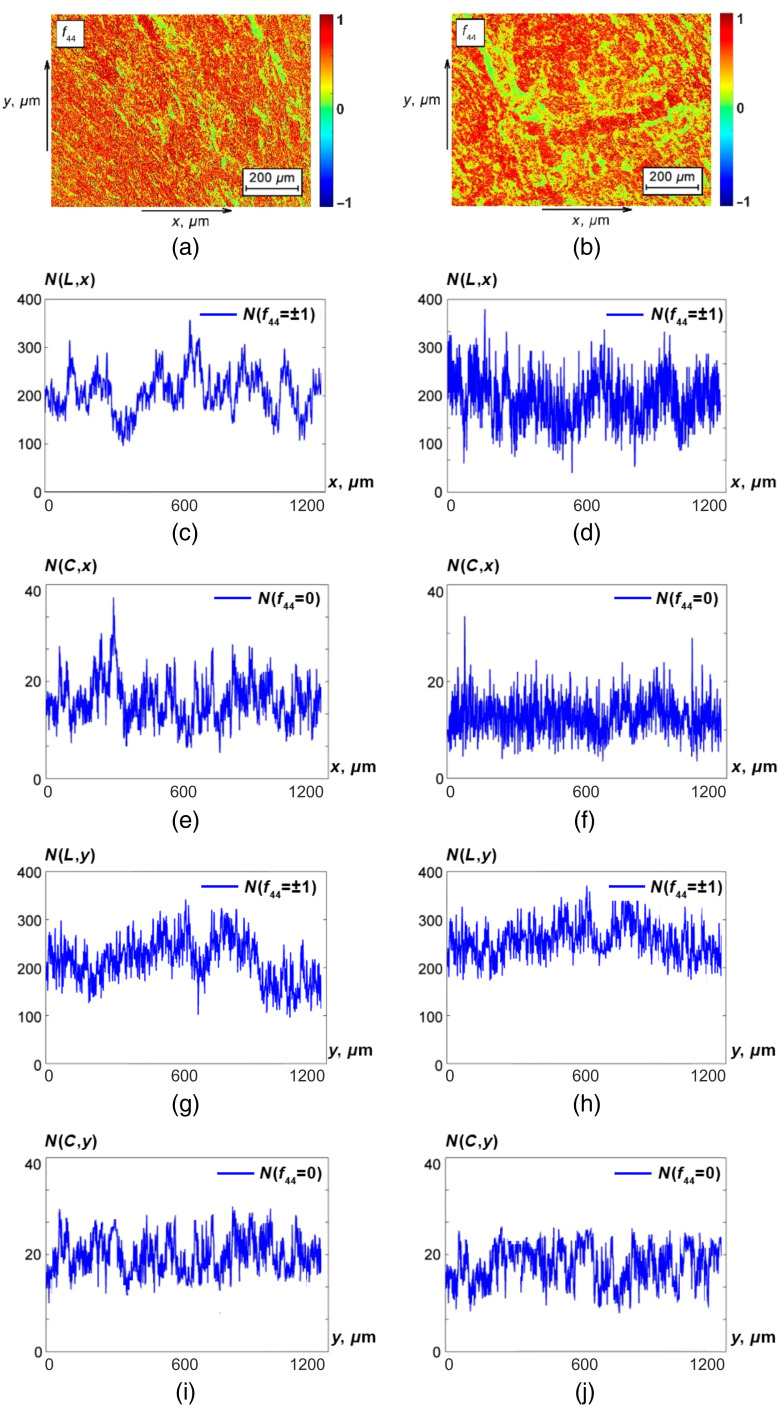
Spatial distributions of characteristic values of the MM images $f_{44}(x,y)$ of histological sections of prostate adenoma (a) and carcinoma (b). Illustrative linear dependences of N(L,x), N(C,x), N(L,y), and N(C,y), respectively, for prostate adenoma (c), (e), (g), (i) and carcinoma (d), (f), (h), (j). See further details in the text.

The results of the polarization-singular study (using MM images of $f_{44}(x,y)$ invariants) of the polycrystalline structure for optically thin histological sections of benign (adenoma) and malignant (carcinoma) prostate tumor tissue samples are shown in Fig. 2. Here, Figs. 2(a) and 2(b) show the MMI $f_{44}(x,y)$ of the adenoma [Fig. 2(a)] and carcinoma [Fig. 2(b)] samples. Figures 2(c)–2(f) show distributions of $N((f_{44}=\pm 1),x)\equiv N(L,x)$ (Figs. 2(c) and 2(d)] and $N((f_{44}=0),x)\equiv N(,x)$ (Figs. 2(e) and 2(f)]. Similar dependencies of the number of characteristic values $f_{44}(x,y)$ obtained for the orthogonal scanning direction (Oy) are presented [Figs. 2(g)–2(j)].

Comparative analysis of the obtained data (Fig. 2) revealed opposite tendencies in changes of distributions N(L) and N(C) during the formation of malignant carcinoma of the prostate regardless of scanning direction (Ox and Oy). The number of characteristic values $f_{44}=0$ decreases [Figs. 2(c) and 2(d) and 2(e) and 2(f)] and the number of characteristic values $f_{44}=\pm 1$ increases (Figs. 2(g) and 2(h) and 2(i) and 2(j)]. Physically, the obtained results can be explained by the fact that malignant necrotic changes in prostate tissue lead to degradation of its polycrystalline birefringent structure.^17^^,^^18^ As a consequence of this process, the “phase-shifting” ability of this layer decreases (δ↓) and the probability of formation of C-polarization states decreases. By contrast, the probability of L-states formation increases, which corresponds to optically isotropic necrotically changed areas of carcinoma tissue.

Quantitatively, these birefringence degradation processes of malignant prostate tumors are illustrated by the results of statistical ($Z_{i}$) and informational (Se,Sp,Ac) analysis of the $f_{44}(x,y)$, $\left\{ \begin{array}{c} N((f_{44}=\pm 1),x)\equiv N(L,x);\\ N((f_{44}=\pm 1),y)\equiv N(L,y) \end{array}\right.$, and $\left\{ \begin{array}{c} N((f_{44}=0),x)\equiv N(C,x);\\ N((f_{44}=0),y)\equiv N(C,y). \end{array}\right.$ These are presented in Tables 3 and 4.

**Table 3:** Statistical informational parameters characterizing the distribution of $f_{44}(x,y)$, $N((f_{44}=\pm 1),x)$, and $N((f_{44}=0),x)$ within both groups of prostate samples.

Parameters	$f_{44}(x,y)$		$N((f_{44}=\pm 1),x)$		$N((f_{44}=0),x)$	
Samples	Adenoma	Carcinoma	Adenoma	Carcinoma	Adenoma	Carcinoma
M	0.31	0.26	237.3	288.9	19.4	16.3
	± 0.019	± 0.017	± 18.8	± 24.7	± 1.2	± 0.91
p	p<0.05		p<0.05		p<0.05	
Se (%)	55.5		66.7		72.2	
Sp (%)	52.8		63.9		69.4	
Ac (%)	54.15		65.3		70.8	
D	0.19	0.14	52.1	59.7	7.8	5.1
	± 0.009	± 0.008	± 3.5	± 4.2	± 0.41	± 0.032
p	p<0.05		p<0.05		p<0.05	
Se (%)	61.1		72.2		77.8	
Sp (%)	58.3		69.4		75	
Ac (%)	59.4		70.8		76.4	

**Table 4:** Statistical informational parameters characterizing the distribution of $f_{44}(x,y)$, $N((f_{44}=\pm 1),y)$, and $N((f_{44}=0),y)$ within both groups of prostate samples.

Parameters	$f_{44}(x,y)$		$N((f_{44}=\pm 1),y)$		$N((f_{44}=0),y)$	
Samples	Adenoma	Carcinoma	Adenoma	Carcinoma	Adenoma	Carcinoma
M	0.31	0.26	214.3	259.9	21.4	17.1
	± 0.019	± 0.017	± 13.7	± 21.4	± 1.1	± 0.88
p	p<0.05		p<0.05		p<0.05	
Se (%)	55.5		68.4		74.3	
Sp (%)	52.8		64.2		70.6	
Ac (%)	54.15		66.3		72.5	
D	0.19	0.14	49.4	57.6	7.2	4.9
	± 0.009	± 0.008	± 3.5	± 4.2	± 0.3.8	± 0.029
p	p<0.05		p<0.05		p<0.05	
Se (%)	61.1		74.8		80.4	
Sp (%)	58.3		71.6		77.2	
Ac (%)	59.4		73.82		78.8	

The analysis of the presented data showed the low efficiency of the traditional MM polarimetry imaging method for discrimination between different types of prostate tumors (54.15%≤Ac(M,D)≤59.5%). At the same time, utilizing the statistical analysis of the distributions of characteristic values (N(L,x) and N(C,x)), the balanced accuracy of differential diagnosis is increased by 10% to 15% (65.3%≤Ac(N(L,C))≤76.4%).

Similar results (within 5% of variation in the value of the balanced accuracy Ac) were obtained using statistical and polarization-singular analysis of the set of other MM invariants $F_{ik}$ (Table 5).

**Table 5:** Operational characteristics of MM invariants and polarization-singularity methods diagnostic power.

MMI	$f_{44}$		$(f_{22}+f_{33})-1$		$\sqrt{\left(f_{42}^{2}+f_{43}^{2}\right)}$		$\sqrt{\left(f_{24}^{2}+f_{34}^{2}\right)}$	
${\mathrm{MMI}}^{*}$	N(±1)	N(0)	N(±1)	N(0)	N(±1)	N(0)	N(±1)	N(0)
Ac(x) (%)	65.3 to 70.8	70.8 to 76.4	67.1 to 72.5	72.8 to 78.4	68.9 to 74.1	64.4 to 70.1	68.9 to 74.1	65.1 to 69.7
Ac(y) (%)	66.3 to 73.2	72.45 to 78.8	69.1 to 75.3	75.5 to 80.3	65.1 to 71.4	70.4 to 76.6	65.8 to 72.1	71.2 to 77.1

**Fig. 3:**
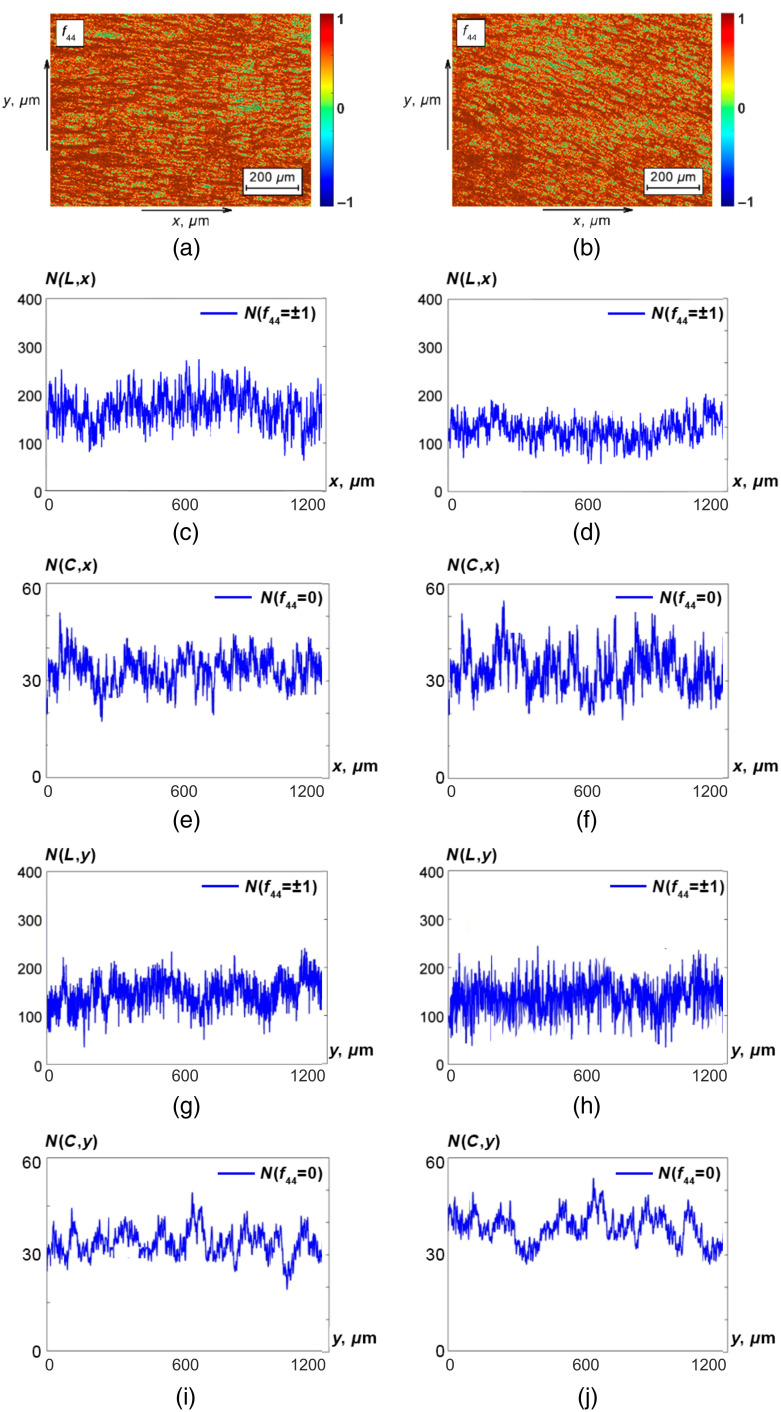
Spatial distributions of characteristic values of the MM images $f_{44}(x,y)$ of histological sections of prostate adenoma (a) and carcinoma (b). Illustrative linear dependences of N(L,x), N(C,x), N(L,y), and N(C,y), respectively, for prostate adenoma (c), (e), (g), (i) and carcinoma (d), (f), (h), (j). See further details in the text.

This result can be related to the fact that all MM images that characterize optical anisotropy of the polycrystalline structure for histological sections of prostate tumor tissue samples are functionalities of a single physical mechanism—phase-shifting capacity of linear birefringence—$F_{ik}(\sigma )$. In addition, for both scanning directions (Ox and Oy), the statistical (M,D) and informational (Ac) parameters of both methods are close enough—the differences between M,D lie within 8% to 15%, and variations of Ac diagnostic accuracy do not exceed 2% to 3%. In addition, the obtained result can be explained by the fact that pathological changes of fibrillar networks of prostate tumor samples are sufficiently azimuthally symmetric. On the other hand, for other tissue types and pathologies, other scenarios of birefringent polycrystalline structure changes can also be realized. For this purpose, we performed an additional set of studies aimed at the differential diagnosis of extragenital endometriosis (group 3 and group 4) by methods of azimuthal-invariant MM polarimetry and polarization-singular MM image analysis (see Fig. 3, Tables 6–8).

**Table 6:** Statistical informational parameters characterizing the distribution of $f_{44}(x,y)$, $N((f_{44}=\pm 1),x)$, and $N((f_{44}=0),x)$ within both groups of uterine samples.

Parameters	$f_{44}(x,y)$		$N((f_{44}=\pm 1),x)$		$N((f_{44}=0),x)$	
Samples	Myoma	Endometriosis	Myoma	Endometriosis	Myoma	Endometriosis
M	0.21	0.16	188.5	141.6	29.1	38.4
	± 0.014	± 0.009	± 11.8	± 9.8	± 1.6	± 2.3
p	p<0.05		p<0.05		p<0.05	
Se (%)	66.3		77.4		81.6	
Sp (%)	62.8		74.8		78.8	
Ac (%)	64.5		76.1		80.2	
D	0.12	0.11	41.3	49.9	11.5	16.3
	± 0.007	± 0.006	± 2.7	± 3.2	± 0.07	± 0.09
p	p<0.05	—	p<0.05		p<0.05	
Se (%)	72.3		81.2		84.8	
Sp (%)	68.4		79.4		80.3	
Ac (%)	70.3		80.3		82.5	

**Table 7:** Statistical informational parameters characterizing the distribution of $f_{44}(x,y)$, $N((f_{44}=\pm 1),y)$, and $N((f_{44}=0),y)$ within both groups of uterine samples.

Parameters	$f_{44}(x,y)$		$N((f_{44}=\pm 1),y)$		$N((f_{44}=0),y)$	
Samples	Myoma	Endometriosis	Myoma	Endometriosis	Myoma	Endometriosis
M	0.21	0.16	153.4	120.8	33.5	46.8.1
	± 0.014	± 0.009	± 9.8	± 7.74	± 2.2	± 3.2
p	p<0.05		p<0.05		p<0.05	
Se (%)	66.3		77.2		79.8	
Sp (%)	62.8		74.9		75.2	
Ac (%)	64.5		76.1		77.5	
D	0.12	0.11	32.6	58.9	14.6	21.2
	± 0.007	± 0.006	± 2.1	± 3.9	± 0.08	± 0.11
p	p<0.05		p<0.05		p<0.05	
Se (%)	72.3		81.4		85.2	
Sp (%)	68.4		77.4		81.6	
Ac (%)	70.4		79.4		83.4	

**Table 8:** Operational characteristics of MM invariants (MMI) and polarization-singularity methods diagnostic power.

MMI	$f_{44}$		$(f_{22}+f_{33})-1$		$\sqrt{\left(f_{42}^{2}+f_{43}^{2}\right)}$		$\sqrt{\left(f_{24}^{2}+f_{34}^{2}\right)}$	
${\mathrm{MMI}}^{*}$	N(±1)	N(0)	N(±1)	N(0)	N(±1)	N(0)	N(±1)	N(0)
Ac(x) (%)	76.1 to 80.3	80.2 to 82.5	78.4 to 83.1	83.1 to 85.3	74.5 to 78.4	78.8 to 80.4	74.1 to 77.8	77.6 to 79.1
Ac(y) (%)	76.1 to 79.4	77.5 to 83.4	73.8 to 76.7	74.1 to 78.7	74.2 to 75.6	73.8 to 76.2	72.9 to 75.4	73.2 to 76.3

Comparative analysis of the obtained data (Fig. 3) revealed different (opposite to Fig. 2) tendencies in N(L) and N( C) distributions changing during uterine endometriosis formation in both scanning directions (Ox and Oy): the number of characteristic values $f_{44}=0$ increases [Figs. 3(c) and 3(d) and 3(g) and 3(h)], whereas the number of characteristic values $f_{44}=1$ decreases [Figs. 3(e) and 3(f) and 3(i) and 3(j)].

The obtained results can be explained by the fact that pathological endometriosis overgrowth of fibrillar networks of connective tissue leads to an increase in the level of linear birefringence.^31^^,^^32^^,^^48^^,^^49^ As a consequence of this process, the “phase-shifting” ability of this layer increases (σ↑), and the probability of the formation of C-polarization states increases as well. By contrast, the probability of forming L states, which correspond to optically isotropic altered regions of the uterine endometrium, decreases.

Analysis of the data presented in Tables 6–8 revealed a slightly higher (∼10%) diagnostic efficiency of myoma and extragenital endometriosis differentiation by azimuthal-invariant MM polarimetry: 64.55%≤Ac(M,D)≤70.35%. Polarization-singular processing of the data obtained provides a further increase in the level of balanced accuracy to the level of 76.1%≤Ac(N(L,C))≤83.4%. Similar results (within 5% variation in the value of the balanced accuracy Ac) were obtained using statistical and polarization-singular analysis of the set of other MM invariants $F_{ik}$ (Table 8). At that, for both scanning directions (Ox and Oy), the differences between M,D increased 12% to 20%, and the variation in the accuracy Ac was 7% to 9%. The obtained results can be explained by the fact that the pathological formation of newly formed fibrillar networks of endometrial connective tissue leads to an increase in structural anisotropy^48^^,^^49^ (linear birefringence), the polarization manifestations of which may be azimuthally asymmetric.

The main factor limiting the accuracy of MM differential diagnosis of pathological changes is the integral averaging of experimentally obtained information about optical anisotropy over all coordinates and geometric dimensions of the morphological structure of biological tissues.^10^^–^^18^ We show (Tables 3–8) that the use of polarization-singular samples from the whole array of MM invariant values provides an increase in the accuracy of differential diagnosis of pathological conditions of the prostate and uterine endometrium tissues by 15% to 20%. It should be noted that the mentioned processes are scale-dependent. Oncological changes that are accompanied by necrotic destruction of morphological structure are localized in large-scale areas of birefringent fibrillary networks of the prostate. By contrast, the formation (growth) of fibrillar networks of connective tissue of the uterine endometrium are localized in small-scale areas. Based on this, we next investigated additional possibilities of scale-selective wavelet analysis [Eqs. (1)–(4)] of distributions of number of characteristic MM invariant values to improve the accuracy of differential diagnostics of biological tissues from different human organs.

**Fig. 4:**
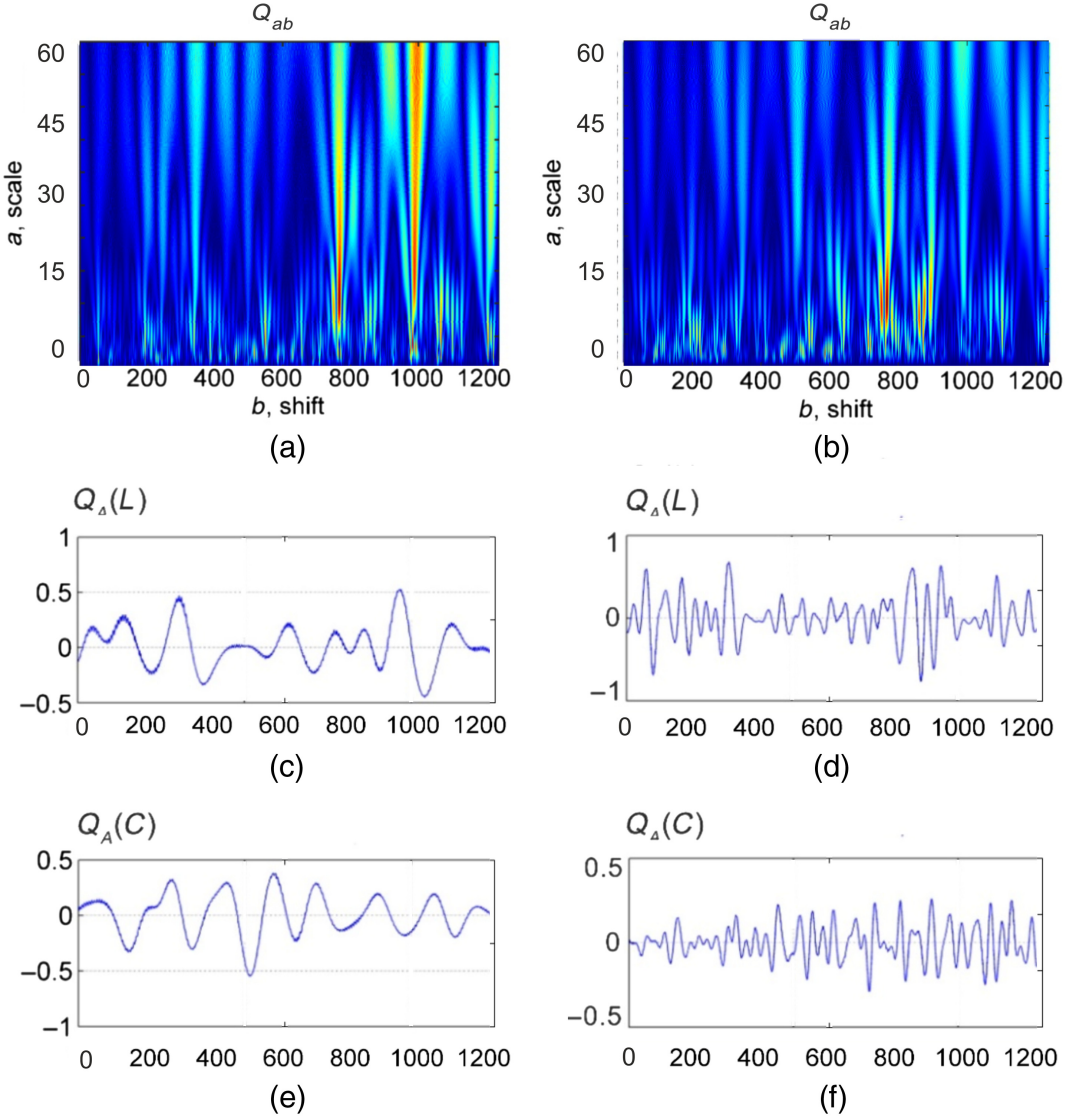
Wavelet transform of distributions N(L,x) and N(C,x) for (a) adenoma and (b) carcinoma tissues. Linear dependencies of the amplitudes of the wavelet coefficients $Q_{A^{*}}(L,b)$ and $Q_{A^{*}}(C,b)$ on the optimal scale $A^{*}$ of the MHAT function $\Omega_{ab}$ of MM image characteristic values $f_{44}(x,y)$ for (c), (e) adenoma and (d), (f) carcinoma tissues, respectively.

**Fig. 5:**
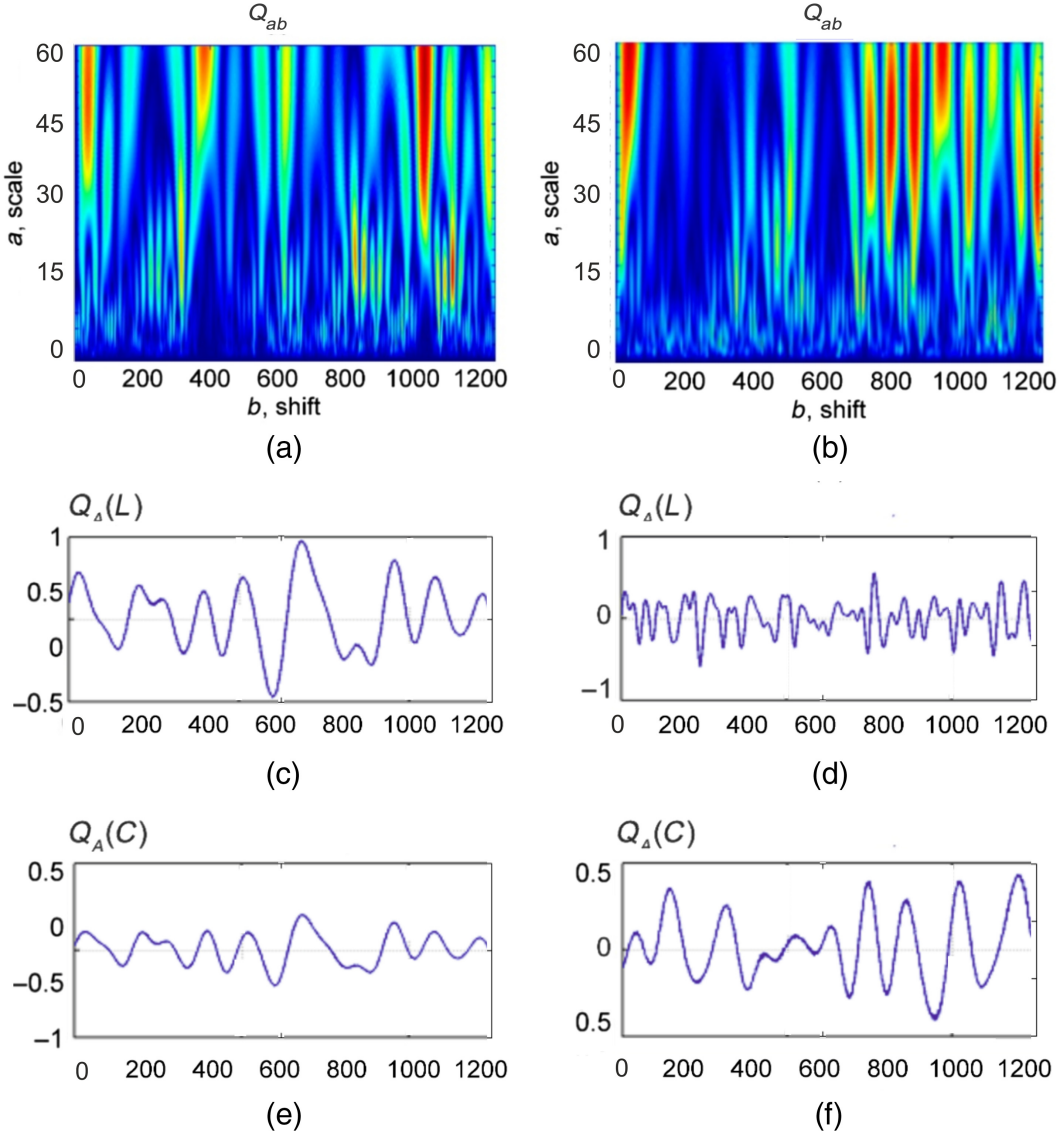
Wavelet transform of distributions N(L,x) and N(C,x) for (a) adenoma and (b) carcinoma tissues. Linear dependencies of the amplitudes of the wavelet coefficients $Q_{A^{*}}(L,b)$ and $Q_{A^{*}}(C,b)$ on the optimal scale $A^{*}$ of the MHAT function $\Omega_{ab}$ of MM image characteristic values $f_{44}(x,y)$ for (c), (e) myoma and (d), (f) endometriosis tissues, respectively.

Figures 4 and 5 show the wavelet transform maps $Q_{ab}$ [Figs. 4, 5(a), and 5(b)] of distributions N(L,x), N(C,x), the linear dependencies of the amplitudes of the wavelet coefficients $Q_{A^{*}}(L,b)$ [Figs. 4, 5(c), and 5(d)] and $Q_{A^{*}}(C,b)$ [Figs. 4(e) and 4(f)] on the optimal scale $A^{*}$ of the MHAT function $\Omega_{ab}$ of MM image characteristic values $f_{44}(x,y)$ for adenoma and carcinoma (Fig. 4) and myoma and endometriosis (Fig. 5) tissues.

A comparative analysis of $Q_{A}(L,b)$ and $Q_{A}(C,b)$ distributions revealed opposite tendencies in the formation of the magnitude and range of variation of their amplitudes $Q_{A}$. First, for the carcinoma tissues, the range of amplitude changes of wavelet coefficients for $Q_{A}(C,b)$ was smaller than that of the adenoma tissues. At the same time, the range of amplitude changes of wavelet coefficients for $Q_{A}(L,b)$ was higher for adenoma tissues than for the carcinoma.

For the endometrium tissues, the range of amplitude changes of wavelet coefficients for $Q_{A}(C,b)$ was higher compared with the myoma tissues. At the same time, the amplitude range of changes of wavelet coefficients for $Q_{A}(L,b)$ was smaller for endometrium tissues than for myoma.

From a physical point of view, the obtained results can be explained by the fact that cancer development leads to the destruction of birefringent large-scale localized domains of the prostate tissue. Thus, in corresponding areas, the value of the phase shifts decreases. In this way, the probability of C -states formation and the number of characteristic values of MM image $f_{44}(x,y)=0$ are reduced. Therefore, for a given scale $A^{*}$, during scanning $Q_{A}(C,b)$, the maximum extrema $Q_{A^{*}}$ of the distributions of the wavelet coefficients are formed. The opposite picture takes place in the wavelet analysis of distributions $Q_{A^{*}}(L,b)$ characterizing the number of L-states corresponding to necrotically changed (almost optically isotropic) areas of adenocarcinoma tissues, which are characterized by values $f_{44}(x,y)=\pm 1$. As a result, for adenocarcinoma samples, the average and dispersion characterized the distributions of wavelet coefficients amplitudes of the number of L-states. The opposite picture takes place for the statistical parameters characterizing the distributions $Q_{A}(C,b)$.

For the pathological growth of fibrillar networks of endometrium connective tissue, the opposite tendencies to that of prostate tissue are realized. Specifically, for myoma samples, the average and dispersion, characterizing the distributions of the amplitudes of the wavelet coefficients of the number of L-states, compared more to the same parameters of the carcinoma samples. The opposite picture takes place for the statistical parameters of prostate tumors samples.

The quantitative data for the distributions $Q_{A}(L,b)$ and $Q_{A}(C,b)$ for prostate and uterine tissues are presented in Tables 9 and 10, respectively.

**Table 9:** Statistical and informational parameters characterizing the distributions $Q_{A}(L,b)$ and $Q_{A}(C,b)$ within both groups of prostate samples.

Parameters	$Q_{A}(L,b)$		$Q_{A}(C,b)$	
Samples	Adenoma	Carcinoma	Adenoma	Carcinoma
M	0.051	0.045	0.012	0.015
	± 0.004	± 0.003	± 0.001	± 0.0012
p	p≺0.05		p≺0.05	
Se (%)	77.8		80.6	
Sp (%)	75		77.8	
Ac (%)	76.4		79.2	
D	0.31	0.25	0.11	0.18
	± 0.021	± 0.018	± 0.006	± 0.008
p	p≺0.05		p≺0.05	
Se (%)	86.1		94.4	
Sp (%)	83.3		91.7	
Ac (%)	84.7		93.05	

**Table 10:** Statistical and informational parameters characterizing the distributions $Q_{A}(L,b)$ and $Q_{A}(C,b)$ within both groups of uterine samples.

Parameters	$Q_{A}(L,b)$		$Q_{A}(C,b)$	
Samples	Myoma	Endometriosis	Myoma	Endometriosis
M	0.048	0.037	0.018	0.025
	± 0.003	± 0.002	± 0.001	± 0.0011
p	p≺0.05		p≺0.05	
Se (%)	83.6		84.8	
Sp (%)	80.2		82.4	
Ac (%)	81.9		83.6	
D	0.27	0.21	0.16	0.23
	± 0.016	± 0.013	± 0.007	± 0.014
p	p≺0.05		p≺0.05	
Se (%)	90.4		98.8	
Sp (%)	87.5		96.6	
Ac (%)	88.9		97.7	

The accuracy of the wavelet analysis of the distributions $Q_{A^{*}}(C,b)$ of MM image characteristic values $f_{44}(x,y)$ in the differentiation of the tumor states for the prostate tissue reaches an excellent quality of Ac(N(C))=93.05%, and for uterine tissue, Ac(N(C))=97.7%.

Thus, the proposed method of wavelet analysis of the characteristic value distributions for MM images of linear birefringence significantly expands the functionality of the traditional polarization mapping of histological sections with minor changes in the optical anisotropy of fibrillar networks.

## 4 Conclusions

In this study, an analytical relationship between the characteristic values of individual matrix elements and polarization singularities of microscopic images of birefringent fibrillar networks of biological tissues were established within the framework of an MM model of phase anisotropy. The elements of MM treated with the wavelet analysis were used to diagnose the local manifestations of localized changes in the magnitude of birefringence of the polycrystalline fibrillar compounds within biological tissue. The statistical analysis of characteristic values of spatial distributions of the obtained MM images demonstrates a high potential for differentiating the benign and malignant states of the prostate and uterine tissues with excellent accuracy.
